# Management of the Adnexal Mass: Considerations for the Family Medicine Physician

**DOI:** 10.3389/fmed.2022.913549

**Published:** 2022-07-05

**Authors:** Brian Bullock, Lisa Larkin, Lauren Turker, Kate Stampler

**Affiliations:** ^1^Main Line Health, Philadelphia, PA, United States; ^2^Lisa Larkin, MD, and Associates, Cincinnati, OH, United States; ^3^Ms. Medicine Healthcare Organization, Cincinnati, OH, United States; ^4^Cincinnati Sexual Health Consortium, Cincinnati, OH, United States; ^5^Einstein Healthcare Network, Philadelphia, PA, United States

**Keywords:** multivariate index assay, biomarkers, ultrasound, simple rules, pelvic mass, ovarian cancer, family medicine

## Abstract

Ovarian cancer is the most deadly gynecological cancer, so proper assessment of a pelvic mass is necessary in order to determine which are at high risk for malignancy and should be referred to a gynecologic oncologist. However, in a family medicine setting, evaluation and treatment of these masses can be challenging due to a lack of resources. A number of risk assessment tools are available to family medicine physicians, including imaging techniques, imaging systems, and blood-based biomarker assays each with their respective pros and cons, and varying ability to detect malignancy in pelvic masses. Effective utilization of these assessment tools can inform the care pathway for patients which present with an adnexal mass, such as expectant management for those with a low risk of malignancy, or referral to a gynecologic oncologist for surgery and staging, for those at high risk of malignancy. Triaging patients to the appropriate care pathway improves patient outcomes and satisfaction, and family medicine physicians can play a key role in this decision-making process.

## Introduction

Pelvic masses are a common gynecological health issue. An estimated 10% of women will undergo surgery for a mass in their lifetime. Despite the high prevalence of adnexal masses, less than 20% of newly discovered pelvic masses will be malignant (1–3). The overall incidence of ovarian cancer is relatively low, accounting for just over 1% of all cancer diagnosis in 2021. However, ovarian cancer carries a high mortality rate and it is prudent to for clinicians to be able to identify which pelvic masses are concerning for malignancy.

At present, there is no recommended screening method for ovarian cancer in a healthy patient population (4). As such, guideline recommendations involve assessing a mass based on its characteristics and the patient’s individual risk factors, once a pelvic mass is discovered, usually beginning with visualizing the mass via imaging. The most common imaging modality used to characterize pelvic masses is transvaginal ultrasonography (TVUS), but they may also be incidentally identified by CT scans or MRI. Unfortunately, all of these imaging methods may fall short in their ability to accurately assess the likelihood of malignancy due to their variable sensitivities and specificities. Of greater clinical significance, imaging findings which are indicative of malignancy often overlap with benign pathologies, resulting in subjective interpretation of risk. Clinicians who deal with these cases less frequently may not have the same level of expertise in making these calls, while experienced oncology and radiology specialists have a high accuracy for assessing masses based on imaging features (5, 6). The presence of indeterminate cases using imaging leaves clinicians facing difficult decisions on proceeding with surgery or referring to a subspecialist for surgery and staging.

Along with imaging challenges, the non-specific symptoms and risk factors of ovarian cancer create difficulties in identifying malignancy, particularly at an early stage. The majority of women with ovarian cancer (75%) are diagnosed at an advanced stage (7). This is mainly due to the asymptomatic nature of early stage disease as well as the non-specific symptoms of late-stage disease. These symptoms are easily confused with those belonging to other pelvic or abdominal ailments. As an additional factor, most women with ovarian cancer do not have a relevant family history. Only 10–12% of cases have an underlying genetic etiology (7). Biopsy of masses is not recommended due to the risk of rupturing the capsule and spreading any potentially malignant cells, which can eliminate a method of identifying potential malignancy from the presence of abnormal cells (8). All of these factors combined make identifying women who are clearly at elevated risk of malignancy difficult.

Practice guidelines support referral of suspected malignant masses to a gynecologic oncologist, citing the improved outcomes associated with referral (9). However, referral to a gynecologic oncologist may not be warranted or desirable in low-risk cases. Gynecologic oncologists are limited in the US (only 13% working outside of large metropolitan centers); so access to these specialists is especially challenging for the approximately 30 million women who live outside of largely populace areas (10, 11).

Blood-based tests can assist practitioners in understanding a patient’s risk of malignancy and guiding management decisions. These blood tests may be used in conjunction with imaging to increase confidence in a clinical impression.

This review describes current non-surgical tests available to family medicine physicians that aid in the detection, risk, diagnosis and treatment of ovarian cancer and the tools which are available for the family medicine physician’s utilization.

## Evaluation Options, Guidelines, and Implications

### First Line of Detection

Within a family medicine setting, pelvic masses may be asymptomatic and discovered incidentally during routine physical exams. Alternatively, patients may present with symptoms concerning for a possible pelvic mass such as irregular vaginal bleeding, bloating, increased abdominal girth, dyspareunia, urinary symptoms, pelvic pain, and abdominal pain. Many of these symptoms are non-specific, which results in a significant number of masses being discovered while treating unrelated conditions (12).

While the primary risk factor for ovarian cancer is age, with approximately 70% of malignancies occurring in patients age 55 or older, there are specific types of ovarian malignancies which primarily occur in patients under 40, or even adolescents (3). As such, it is recommended that all pelvic masses be assessed for risk of malignancy (1). Generally, the first line means of characterization and risk assessment for these masses, and more typically symptomatic ones, is transvaginal ultrasound (TVUS), which can help stratify masses as benign or malignant based on morphology alone. The American College of Obstetricians and Gynecologists (ACOG) recommends TVUS, or abdominal ultrasound in cases wherein the pelvic structures are obscured, distorted, or TVUS cannot be performed, as the first line of characterization for suspected pelvic masses (1).

### Ultrasound

Pelvic ultrasound has been examined as a potential screening modality for ovarian cancer. In large prospective clinical trials of asymptomatic women carried out by The University of Kentucky Ovarian Cancer Screening Project and The Prostate, Lung, Colorectal and Ovarian (PLCO) Cancer Screening Trial, TVUS was found to be fairly sensitive with regards to identifying potential malignancies based on abnormal morphologies. However, it was less able to distinguish between benign and cancerous masses, resulting in a poor positive predictive value (13, 14).

Another consideration is characterization of these masses is dependent on the subjective interpretation of an ultrasonographer (15). Physicians in primary care settings have limited access to in-house diagnostic equipment and lack radiologists or other professionals with extensive ultrasonography expertise. As a result, physicians are often dependent on external radiology groups for diagnostic imaging services, and in resource-constrained areas, these services may be limited (15). The sensitivity of TVUS can vary significantly depending on the experience of the interpreter. A prospective trial consisting of 199 women with adnexal masses (37.7% prevalence) demonstrated a TVUS sensitivity of 96.2% and a specificity of 96.3% for experienced sonographers using subjective assessment. For less experienced sonographers, however, the sensitivity was 72.4% and the specificity 88.8%, a significant difference in area under the curve (AUC) value (5, 6). Because of this ambiguity, ultrasound systems and specific assessment criteria have been created to help aid physicians in risk stratification of pelvic masses.

The Society of Radiologists in Ultrasound (SRU) consensus statement, popular in North America, is helpful in determining a clinical care pathway for adnexal masses based on TVUS results (16). This statement describes the recommendations for pre- and post-menopausal masses based on their size and physical features. Small simple cysts with no Doppler color flow may not require additional follow up, as these are likely to be simple physiologic cysts which are associated with normal ovulation, and are likely to resolve within 6 months. Larger cysts, or cysts with more complex morphology may require regular monitoring, or surgical intervention. The statement also describes features which are indicative of potential malignancy, such as multiple septations and nodules, for which surgical intervention is recommended.

The American College of Obstetricians and Gynecologists (ACOG) has also released guidelines for assessing adnexal masses based on TVUS features, summarized below:

1. Benign: Simple cysts under 10 cm.
2. Malignant: Solid Mass, Separations > 3 mm, Mural Nodules, Papillary Excrescences, Ascites (Free Fluid).
3. Indeterminate: Complex Masses of any size, Simple cysts > 10 cm (1).

Using the criteria laid out by either the SRU or ACOG, many masses identified by ultrasound fall into a category that is neither clearly benign nor clearly malignant and are ultimately labeled as “indeterminate.” Indeterminate masses make up 23.9% of pelvic masses, based on a 2018 study to assess their prevalence (17). Indeterminate masses are problematic clinically, particularly given the subjective nature of interpreting ultrasound findings. Sonographer expertise is once again a factor here, as more experienced sonographers were more likely to be able to make conclusive diagnoses from TVUS results than those with less experience (5).

### Ultrasound Systems

#### International Ovarian Tumor Analysis

The international ovarian tumor analysis (IOTA) Simple Rules system uses a set of 10 sonographic features to determine risk of malignancy, laid out in Table 1 (18).

**TABLE 1:** A list of the IOTA Simple Rules ultrasonography features for classifying pelvic masses.

IOTA simple rules features (18)	
Benign	Unilocular cyst
	Presence of solod components < 7 mm
	Presence of acoustic shadows
	Smooth multilocular tumor < 10 cm
	No blood flow (color score 1)
Malignant	Irregular solid tumor
	Presence of ascites (free fluid in abdomen)
	≥4 papillary structures
	Irregular multilocular tumor ≥ 10 cm
	Strong blood flow (color score 4)

Masses having only benign features are classified as benign, whereas masses with only malignant features are classified as malignant. Masses with a combination of benign and malignant features, or which have none of the listed features are classified as indeterminate.

A metanalysis demonstrated that this system exhibited a 93% sensitivity and 81% specificity for detection of malignancy in postmenopausal women (19). The IOTA Simple Rules are popular and readily used in Europe due to their ease. In 2016, the American College of Obstetrician and Gynecologists integrated the Simple Rules into their clinical guideline on the evaluation and management of pelvic masses (1). Despite their simplicity, the Simple Rules were reviewed in 697 patients with 764 pelvic masses and an indeterminate classification was assigned to one-third of the total malignancies (20, 21).

Ultrasound systems like IOTA Simple Rules have been found to be more reliable compared to subjective assessment by an inexperienced sonogropher (5).

#### Ovarian-Pelvic Reporting and Data System

A new system designed to decrease ambiguity and increase the consistency in imaging results, ovarian-pelvic reporting and data system (O-RADS), is comprised of 6 categories (22).

O-RADS 0—incomplete evaluation
O-RADS 1—normal premenopausal ovary
O-RADS 2—almost certainly benign category (<1% risk of malignancy)
O-RADS 3—lesions with low risk of malignancy (1% to < 10%)
O-RADS 4—lesions with intermediate risk of malignancy (10% to < 50%)
O-RADS 5—lesions with high risk of malignancy (> 50%).

O-RADS can be used with TVUS or MRI (23). When utilizing this system, one large study by Cao et al. found that 31.3% of examinations for O-RADS Ultrasound fall into Categories 3 or 4, which can lead to the same problem of appropriately triaging an indeterminate result that other imaging systems have. However, in the same study, when classifying O-RADS Categories 2 and 3 as low risk, and Categories 4 and 5 as high risk, reported a sensitivity and specificity of 98.7 and 83.2%, respectively (24).

At the time of this publication, O-RADS is not widely used in the United States, nor is it present in ACOG guidelines.

### Computed Tomography and Positron Emission Tomography

Computed tomography (CT) and positron emission tomography (PET) scans appear to be the least capable of fully characterizing a pelvic mass. This imaging modality can correctly identify characteristics of advanced disease such as septated cysts and masses with solid components or ascites, but prove to be less accurate in identifying and characterizing early stage, low-grade, and borderline tumors. CT and PET scans are rarely used as the first modality in assessing a pelvic mass for these reasons, and are not recommended as a first line of detection and characterization by ACOG (25). Per ACOG guidelines, the best use of CT is to examine the abdomen for potential metastases in high-risk cases (1).

Additionally, a logistical challenge with CT/PET is limited access of the technology in rural areas and the cost of testing which may make it financially prohibitive to some patients (26).

### Magnetic Resonance Imaging

MRI may be the most accurate imaging technology available for determining the nature of a pelvic mass (27, 28). In a recent multicenter cohort study, MRI had a sensitivity of 93% and a specificity of 91% for pelvic masses that were otherwise characterized as indeterminate by ultrasound (29). MRI is not recommended as a first line detection and assessment modality in ACOG guidelines, instead supporting its use in cases that are not easily characterized via TVUS (1).

Limitations of this approach include patient concern about claustrophobia, time and cost (30, 31). Additionally, much like CT, MRI technology may not be found in rural areas because of cost and use limitation (32, 33).

### Biomarker-Based Risk Assessment Tests

It is not uncommon for imaging to result in inconclusive or indeterminate risk stratification. In those cases, biomarker-based tests may be an additional method to further assess an adnexal mass patient’s risk of malignancy. Below is a summary of commonly used biomarker-based assays which are currently available and recommended for use in clinical management guidelines.

#### Cancer Antigen 125-II

CA125-II is a protein expressed on the surface of many different cell types that undergo metaplastic differentiation. It can be elevated in the serum of individuals with a number of different ailments unrelated to cancer, including endometriosis, pelvic inflammatory disease, and inflammatory bowel disease (34–36). While it has been routinely used in evaluation of pelvic masses to determine risk of malignancy, the test is not FDA-cleared for preoperative evaluation, is not covered by Medicare, and has debatable utility as an ovarian cancer risk assessment tool (37). CA125-II is elevated in approximately 80% of patients with epithelial ovarian cancer but in only 50% of patients with stage I disease (38); its ability to assess early stage malignancy is limited. A screening trial found that CA125-II sensitivity for early stage malignancies was only 40% (39).

As an additional consideration, CA125-II is frequently unable to detect certain histologic subtypes of ovarian cancer, notably mucinous carcinomas and many non-epithelial malignancies (40). A recent analysis of 2,305 patients with adnexal masses who had low-risk or “normal” CA125-II values found 104 malignancies. Mucinous carcinomas accounted for approximately 25% of these malignancies, with non-epithelial cancers (germ cell tumors and sex-cord stromal tumors primarily) accounting for another 33% (41). This is clinically significant. Mucinous carcinomas generally have a favorable prognosis when discovered and treated at an early stage, but one that is comparatively worse than other epithelial subtypes when discovered at an advanced stage (42).

Elevated CA125-II is included by the Society of Gynecologic Oncology and the American College of Obstetrics and Gynecology (ACOG) as criteria for referral of a patient with a pelvic mass to a gynecological oncologist, though it is not approved for use as a standalone diagnostic or screening tool. This criteria does not specify a threshold for what constitutes as elevated in premenopausal patients, leading to subjectivity in referrals (41).

#### Risk of Malignancy Algorithm

The Risk of Malignancy Algorithm (ROMA^§^ *;* Fujirebio Diagnostics) is an FDA-cleared test combining tumor markers CA125-II and Human Epididymis Protein 4 (HE4) with menopausal status to assign high or low risk scores. This test was used in a prospective multicenter study involving 531 patients and correctly assigned 93.8% of ovarian cancer patients into the high-risk group (43). However, several investigations suggest that ROMA is no better than CA125-II alone, with no significant difference in sensitivity and specificity between the two. These studies found that adding HE4 to CA125 in the ROMA algorithm did not significantly improve cancer detection rates. In some cases, the addition of HE4 caused a slight, non-significant decrease in detection for certain demographics and cancer subtypes compared to CA125 alone (44, 45).

#### Multivariate Index Assay and Multivariate Index Assay, Second Generation

Cleared by the FDA in 2009 for use in women with a pelvic mass planned for surgery, MIA (Trade name OVA1; Aspira Women’s Health, Austin, TX) is the first protein-based Multivariate Index Assay (MIA). MIA is a qualitative serum test that combines the results of 5 immunoassays (CA125-II, prealbumin, apolipoprotein A-1, β2 microglobulin, and transferrin) into a single numerical score that can indicate a higher risk of malignancy.

Results from MIA demonstrate greater sensitivity compared to CA125-II. In an investigation of 590 women with pelvic masses, MIA detected 93% (95% CI: 87.4–95.7) of malignancies in all stages and menopausal status compared to 77% (95% CI: 69.9–82.8) identified by CA125-II alone (46). Additionally, MIA detected 67.6% of malignancies missed by CA125-II (41).

Evaluation of MIA across races demonstrated performance differences compared with CA125-II (47). A number of independent studies have shown that CA125-II levels are lower in non-Caucasian women (48–51). Black women in the United States also have lower incidence of high grade epithelial ovarian histology than Caucasian women (52). These two factors could cause CA 125-II to underperform in non-Caucasian populations. Sensitivity of MIA in Black women was 79% compared to a sensitivity of 62% with CA 125-II using the Dearking modified cutoffs of 67 U/mL for premenopausal women and 35 U/mL for postmenopausal women (47, 53). Similar data was seen in comparison to ROMA in Black women, with the sensitivity of ROMA at 54% (54). While MIA still performs more poorly in Black women compared to Caucasian women due to the inclusion of CA125-II in its algorithm, the inclusion of additional biomarkers enables it to pick up some malignancies missed by CA125-II alone (41, 47).

Clinical concerns about the lower specificity of MIA resulting in excessive false positives led to the development of a second-generation test, MIA2G (OVERA; Aspira Women’s Health, Austin, TX), that was cleared by the FDA in 2016. MIA2G increased the specificity to 69%. MIA2G was further evaluated in the MIA prospective cohort as previously described (46). Sensitivity for detection of malignancy was equivalent to MIA at 91.3% overall and 90.3% in premenopausal women. Specificity with MIA2G for the group overall was 69.1%, 15% higher than MIA (55).

ROMA and MIA are included in the ACOG guidelines as more sensitive alternatives to CA125, and risk assessment tools to determine the need for referral to a specialist, and like CA125, they are not intended to be used as screening or standalone diagnostics (1).

#### Subtype-Specific Biomarkers

Most ovarian cancers fall under the category of epithelial malignancies, which arise from the ovarian epithelium. These malignancies typically occur in post-menopausal patients, with risk of malignancy being correlated strongly with age, and the majority result in elevated serum CA125, particularly in advanced stage disease (3).

However, there are rarer subtypes which arise from different tissued in the ovary, and are more common in younger patients. They typically do not result in CA125 overexpression, and therefore other biomarkers to assess risk are recommended for these subtypes (1).

Malignant germ cell tumors are much less common than epithelial ovarian malignancies, accounting for < 5% of ovarian cancers, but they have a significantly different biomarker expression profile. ACOG recommendations include 3 biomarker assays to assess risk for these cancer subtypes: Alpha-fetoprotein (AFP), Human chorionic gonadotropin (β-HCG), and Lactic dehydrogenase (LDH). Each is elevated in different types of germ cell tumors, as demonstrated in Table 2 (56). Sex cord stromal tumors are another rare subtype of ovarian malignancy, and most occur in adolescents, excepting adult granulosa cell tumors, which typically present in patients who have recently gone through menopause. These are characterized by the overproduction of sex hormones (androgens or estrogens). ACOG recommends inhibin B as a biomarker to assess risk for adult granulosa cell tumors, as it is elevated in approximately 85% of these (1, 57, 58).

**TABLE 2:** Biomarkers for non-epithelial ovarian malignancies.

Type of non-epithelial malignancy	Biomarker elevated? (1, 56–58)			
	
	AFP	LDH	β -HCG	Inhibin B
Choriocarcinoma	No	Sometimes	Yes	No
Dysgerminoma	No	Yes	Sometimes	No
Embryonal carcinoma	Sometimes	Sometimes	Sometimes	No
Immature teratoma	Sometimes	Sometimes	No	No
Yolk sac tumor	Yes	Yes	No	No
Adult granulosa cell tumor	No	No	No	Yes

### Management of Masses

#### Expectant Management

With utilization of the above stratification tools, a family physician can help triage a woman with an adnexal mass to the appropriate provider. In cases where the mass is very likely to be benign, expectant management can be safely considered. Biomarkers can help to improve prediction that a mass is benign, and a patient can undergo conservative monitoring as opposed to proceeding with surgical intervention immediately. This is reflected in the guidelines, which recommend observation of masses which are likely to be benign (1).

There is a growing body of data to support this strategy. A review of 2,870 septated cysts concluded that septated cystic ovarian tumors without solid areas or papillary projections have a low risk of malignancy and can be followed sonographically without surgery (59). Additionally, the risk of malignancy in unilocular (non-septated) cysts was found to be even lower in the UK Collaborative Trial of Ovarian Cancer Screening, which consisted of 48,053 post-menopausal women with 2,531 unilocular cysts. The risk of malignancy in this group was 00.4% (*n*/*N* = 9/2,531) and, as such, the authors concluded that expectant management for women with simple cysts is exceptionally low-risk as these cysts are largely either stable or self-resolving within the span of a few years (9).

More recently, the IOTA5 trial, a prospective international multicenter study on adnexal mass patients, reported that, out of the 3,144 subjects undergoing expectant management on the basis of benign ultrasound findings, 1,919 had spontaneously resolved within 2 years, with very low incidence of invasive malignancy, or complications such as torsion or rupture (60).

Another study, which excluded subjects with elevated tumor markers, consisted of 1,363 women over the age of 50 with complex masses < 6 cm. 30 borderline tumors or malignancies were found over the course of the trial, all within 7 months of initial discovery of the mass. This demonstrated that low tumor markers are a good predictor of benign pathology for small complex masses (7).

#### Referral and Surgical Intervention

Per guideline recommendations, patients who have findings which are highly suggestive of potential malignancy, such as large solid components, high blood flow, or ascites (free fluid) via TVUS benefit greatly from referral to a gynecologic oncologist (1). These specialists provide more optimal surgical staging and debulking for ovarian cancer patients, which leads to increased survival (61). Inconclusive or indeterminate masses may still be appropriate candidates for referral to gynecologic oncology dependent on additional factors, such as the results of follow-up tests, the patient’s personal risk factors, availability of resources, and confidence in results (9).

However, for patients with a mass which is not likely to be malignant, but may be problematic for the patient in other ways due to size or symptomology, surgical intervention may still be warranted. While many simple cysts will resolve on their own given time, complex adnexal masses with benign pathology may persist for years (62). In these cases, given the very low risk of malignancy, it is generally unnecessary to refer the patient to gynecologic oncology, as a general gynecologist can safely perform the surgery.

Women with ovarian cancer who seek care from a gynecologic oncologist live longer. Several other factors have played a role in the reported survival benefit in women treated with a gynecologic oncologist. Also, gynecologic oncologist-assisted surgery yielded in more comprehensive and guideline-adequate surgeries. Gains in perioperative care and increasingly aggressive surgical goals for debulking, as well as breakthroughs in both upfront chemotherapy and therapeutic medicines for recurring illness, led to national improvements (63).

Patients with ovarian cancer who may not be candidates for surgery can now receive Neoadjuvant Chemotherapy (NACT) and interval debulking surgery. Receiving a few cycles of NACT may result in a significant tumor reduction and, in the case of inoperable patients, may allow them to undergo surgery, potentially transforming their prognosis (64).

Since the period of recurrence has been delayed, the disease’s recurrence appears to be unavoidable, especially in the advanced stages, raising the question of the best recurrence treatment. Therefore, need for personalized therapy arises based on patient’s characteristics (65).

## Clinical Considerations and Recommendations

**FIGURE 1:**
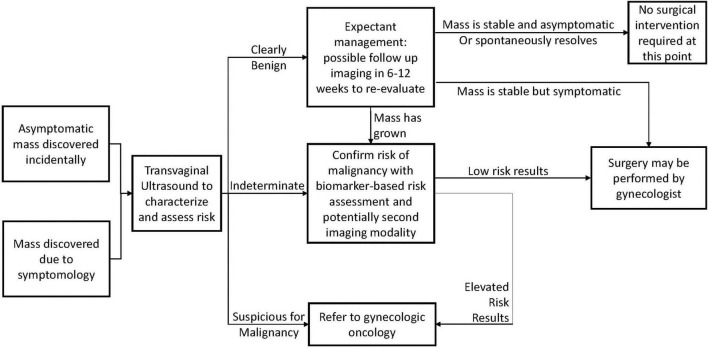
A general care pathway for adnexal masses discovered in a family medicine setting.

Within a family medicine setting, many pelvic masses will be discovered incidentally during routine examinations, and may be entirely asymptomatic. For these, and for symptomatic masses, the general care pathway is presented in Figure 1. ACOG Guidelines recommend characterizing the mass via transvaginal ultrasound to determine the presence of any morphological features indicative of malignancy (1).

The results of this determine the action required by the physician. If the mass presents with features clearly associated with benign pathology, the patient can be observed and re-evaluated after a period of time. After observation, the mass may have spontaneously resolved, or it may have remained stable, at which point it may be possible to determine that no surgical intervention is necessary. Surgery may be warranted if the mass is symptomatic and unresponsive to non-invasive treatments, and low-risk surgeries of this nature may safely be carried out by general gynecologists, and do not require referral to a specialist for treatment.

If the initial TVUS findings were indeterminate, or if a mass has grown larger after observation, it is recommended to use a follow-up risk assessment modality. This may be another imaging technique such as MRI, or it could be a biomarker-based risk assessment tool. A summary of available modalities, their guideline-recommended use, and their performance is presented in Table 3.

**TABLE 3:** Summary of sensitivity and specificity for common methods of pelvic mass risk assessment.

	Imaging				Biomarker-based Assays			
		
	TVUS (Subjective Assessment)* (67)	PET/CT (68)	MRI (69)	IOTA SR** (18, 67)	CA125*** (70, 71)	ROMA (44)	MIA (OVA1) (72)	MIA2G (Overa) (55)
Guideline-recommended use (1)	Initial assessment and characterization	Additional characterization of high risk cases	Characterization of indeterminate masses	Initial assessment and characterization	Aid in risk assessment, guide decision to refer to gynecologic oncologist			N/A, not in 2016 ACOG guidelines
Sensitivity								
All malignancies	73.0–100.0	58.0	91.0	91.0	77.0	84.9	92.2	91.3
Early stage	−	−	−	66.0	50.0	75.0	91.9	88.6
Pre-menopausal	−	−	−	89.0	62.5	67.5	89.5	90.3
Post-menopausal	−	−	−	92.0	80.1	90.8	93.3	91.8
Specificity								
All malignancies	43.0–99.0	76.0	91.0	91.0	93.8	79.7	49.4	69.1

*Range from meta-analysis. **Pooled value from meta-analysis. Indeterminate results had subjective assessment applied. ***Study PI used age </≥ 50 rather than menopausal status. Cutoff value was 35 U/mL for both age groups.

If these confirm low-risk results, surgical intervention is generally recommended as a precaution in most cases, but it is considered safe to be performed by a general gynecologist.

If the initial TVUS, or any secondary assessments for indeterminate cases indicate suspicion of malignancy, referral to a specialist such as a gynecologic oncologist is recommended. Surgical intervention by a gynecologic oncologist according to guideline standards significantly improves outcomes, though a minority of ovarian cancer patients are operated on by these specialists at present (9).

## Conclusion

The family medicine physician plays an instrumental role in helping to identify and appropriately manage a newly discovered pelvic mass. The tools reviewed in this article may assist the frontline physician in identifying women who may truly benefit from referral to a gynecologic oncologist while triaging those who are likely to be benign and can be safely managed by a general gynecologist. For practitioners outside of large city centers, where imaging resources and access to sub-specialists may be limited, the availability of low cost, non-invasive risk assessment tools may be invaluable, particularly when ultrasound findings are indeterminate. Newer biomarker-based risk assessment tools have higher sensitivity compared to older, single-biomarker assays, specifically in early stage disease where intervention makes a critical difference in survival odds. These tests, used in conjunction with imaging, can aid family medicine providers to more accurately, confidently, and rapidly determine if a patient presenting with a pelvic mass is at high risk for ovarian cancer, and referral to a gynecologic oncologist is indicated.

## Author Contributions

All authors listed have made a substantial, direct, and intellectual contribution to the work, and approved it for publication.

## Conflict of Interest

KS has been a consultant for Aspira Women’s Health, Inc., and is currently employed by Einstein Healthcare Network. The remaining authors declare that the research was conducted in the absence of any commercial or financial relationships that could be construed as a potential conflict of interest.

## Publisher’s Note

All claims expressed in this article are solely those of the authors and do not necessarily represent those of their affiliated organizations, or those of the publisher, the editors and the reviewers. Any product that may be evaluated in this article, or claim that may be made by its manufacturer, is not guaranteed or endorsed by the publisher.
